# Ecosystem Engineering by Plants on Wave-Exposed Intertidal Flats Is Governed by Relationships between Effect and Response Traits

**DOI:** 10.1371/journal.pone.0138086

**Published:** 2015-09-14

**Authors:** Maike Heuner, Alexandra Silinski, Jonas Schoelynck, Tjeerd J. Bouma, Sara Puijalon, Peter Troch, Elmar Fuchs, Boris Schröder, Uwe Schröder, Patrick Meire, Stijn Temmerman

**Affiliations:** 1 Department Ecological Interactions, Federal Institute of Hydrology, Koblenz, Germany; 2 Geoinformation in Environmental Planning Lab, Technische Universität Berlin, Berlin, Berlin, Germany; 3 Department of Biology, Ecosystem Management Research Group, University of Antwerp, Wilrijk, Belgium; 4 Centre for Estuarine and Marine Ecology, Royal Netherlands Institute for Sea Research, Yerseke, The Netherlands; 5 UMR 5023 LEHNA, CNRS, Université Lyon 1, ENTPE, Villeurbanne, France; 6 Department of Civil Engineering, Ghent University, Ghent, Belgium; 7 Institute of Geoecology, Environmental Systems Analysis, Technische Universität Braunschweig, Braunschweig, Germany; 8 Berlin-Brandenburg Institute of Advanced Biodiversity Research (BBIB), Berlin, Germany; 9 Department Vegetation Studies & Landscape Management, Federal Institute of Hydrology, Koblenz, Germany; Centro de Investigacion Cientifica y Educacion Superior de Ensenada, MEXICO

## Abstract

In hydrodynamically stressful environments, some species—known as ecosystem engineers—are able to modify the environment for their own benefit. Little is known however, about the interaction between functional plant traits and ecosystem engineering. We studied the responses of *Scirpus tabernaemontani* and *Scirpus maritimus* to wave impact in full-scale flume experiments. Stem density and biomass were used to predict the ecosystem engineering effect of wave attenuation. Also the drag force on plants, their bending angle after wave impact and the stem biomechanical properties were quantified as both responses of stress experienced and effects on ecosystem engineering. We analyzed lignin, cellulose, and silica contents as traits likely effecting stress resistance (avoidance, tolerance). Stem density and biomass were strong predictors for wave attenuation, *S*. *maritimus* showing a higher effect than *S*. *tabernaemontani*. The drag force and drag force per wet frontal area both differed significantly between the species at shallow water depths (20 cm). At greater depths (35 cm), drag forces and bending angles were significantly higher for *S*. *maritimus* than for *S*. *tabernaemontani*. However, they do not differ in drag force per wet frontal area due to the larger plant surface of *S*. *maritimus*. Stem resistance to breaking and stem flexibility were significantly higher in *S*. *tabernaemontani*, having a higher cellulose concentration and a larger cross-section in its basal stem parts. *S*. *maritimus* had clearly more lignin and silica contents in the basal stem parts than *S*. *tabernaemontani*. We concluded that the effect of biomass seems more relevant for the engineering effect of emergent macrophytes with leaves than species morphology: *S*. *tabernaemontani* has avoiding traits with minor effects on wave attenuation; *S*. *maritimus* has tolerating traits with larger effects. This implies that ecosystem engineering effects are directly linked with traits affecting species stress resistance and responding to stress experienced.

## Introduction

Ecosystem engineering has been a key concept for the last 20 years, elucidating how organisms change their abiotic environment and how this feeds back to the biota [1]. Ecosystem engineers construct new niches in ecosystems with strong impacts on ecosystem structure and functioning [1–7]. That in turn has a bearing on ecosystem services [8, 9] and approaches to conservation and restoration in a wide range of ecosystem types [10, 11].

Creating conditions that favour plant growth [12, 13], plants attenuate waves on tidal flats as an ecosystem function [14–16], and the effect of this ecosystem engineering likely varies according to plant functional traits [17]. Traits such as stem density, biomass and flexibility/rigidity determine the ecosystem engineering capacity (EEC) [18–20]. To understand how these traits influence the EEC of different species [21], a fundamental requirement is to clarify which species’ traits respond to the environment (response traits) and which species’ traits determine the effects of plants on ecosystem functions [22, 23]. Traits providing high stress resistance often coincide in effect and response [24]. A better knowledge of these relationships is also crucial so as to understand why different species that occur in the same habitat have a different capacity to deal with abiotic stress and have a different EEC to modify abiotic stress levels, which construct niches with different species distribution patterns [20].

To address the interaction of effect and response traits and the EEC, we used pioneer plants that colonize estuarine tidal flats as a model system. Besides currents, environmental drivers such as waves make estuarine and riverine habitats mechanically stressful environments in which plants can grow. However, plants which were able to establish and grow under these stressful conditions are often autogenic ecosystem engineers, having the effect of reducing hydrodynamic forces and related stress [18, 25]. The stress results from hydrodynamic forces that induce drag forces upon the submerged plant [26, 27] and lead to sediment scour around the stems [28–30]. We would like to find out which traits of these plants have an effect on the species’ capacity for experienced stress (response traits) and which traits determine their EEC.

Plants can avoid stress, or they can tolerate it [31, 32], both are stress resistance strategies [32]. For instance, flexible stems which bend easily are a common trait enabling plants to avoid hydrodynamic stress and are typically found in submerged vegetation [33, 34]. On the contrary, species that are able to grow emergently have more structural rigidity [35] which is regarded as a stress tolerance strategy. This flexibility/rigidity balance can be determined by the balance between cellulose, lignin and silica incorporation [36–38]. These plant traits are species dependent and likely to reflect a cost-benefit trade-off between energy and/or material investment (costs) and survival success by avoiding or tolerating a specific stress level (benefits) [18].

**Fig 1:**
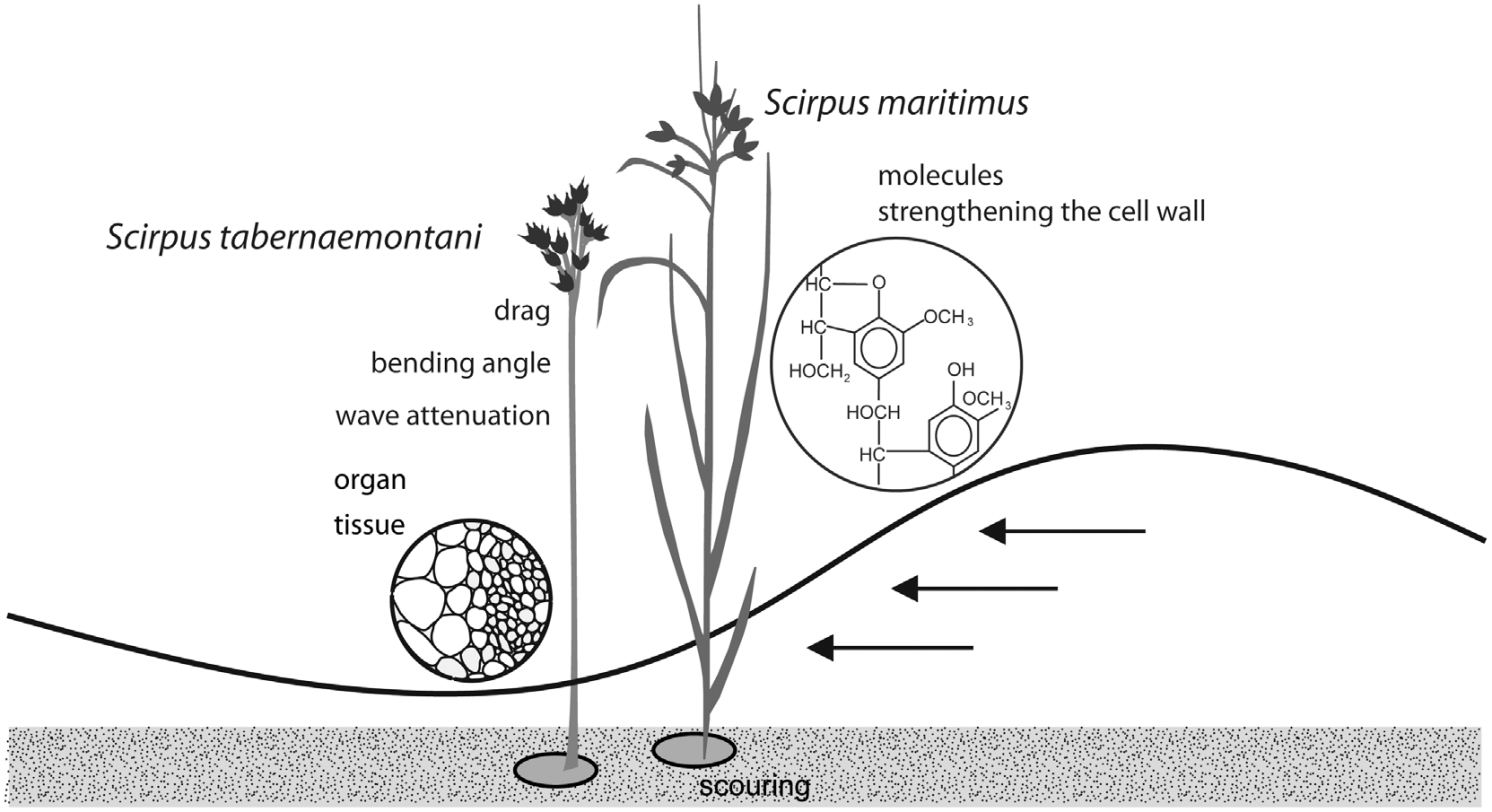
Analyzed traits of two marsh pioneers and their response parameters to wave impact. The molecule traits silica, lignin, and cellulose strengthen the cell wall. Tensile and bending properties of the stem tissue were investigated taking into account the cross-sectional area of the stem organ. Drag, scouring, and the irreversible bending angle of the plants were measured as response parameters to wave impact. The amount of wave attenuation was determined using plant patches.

**Fig 2:**
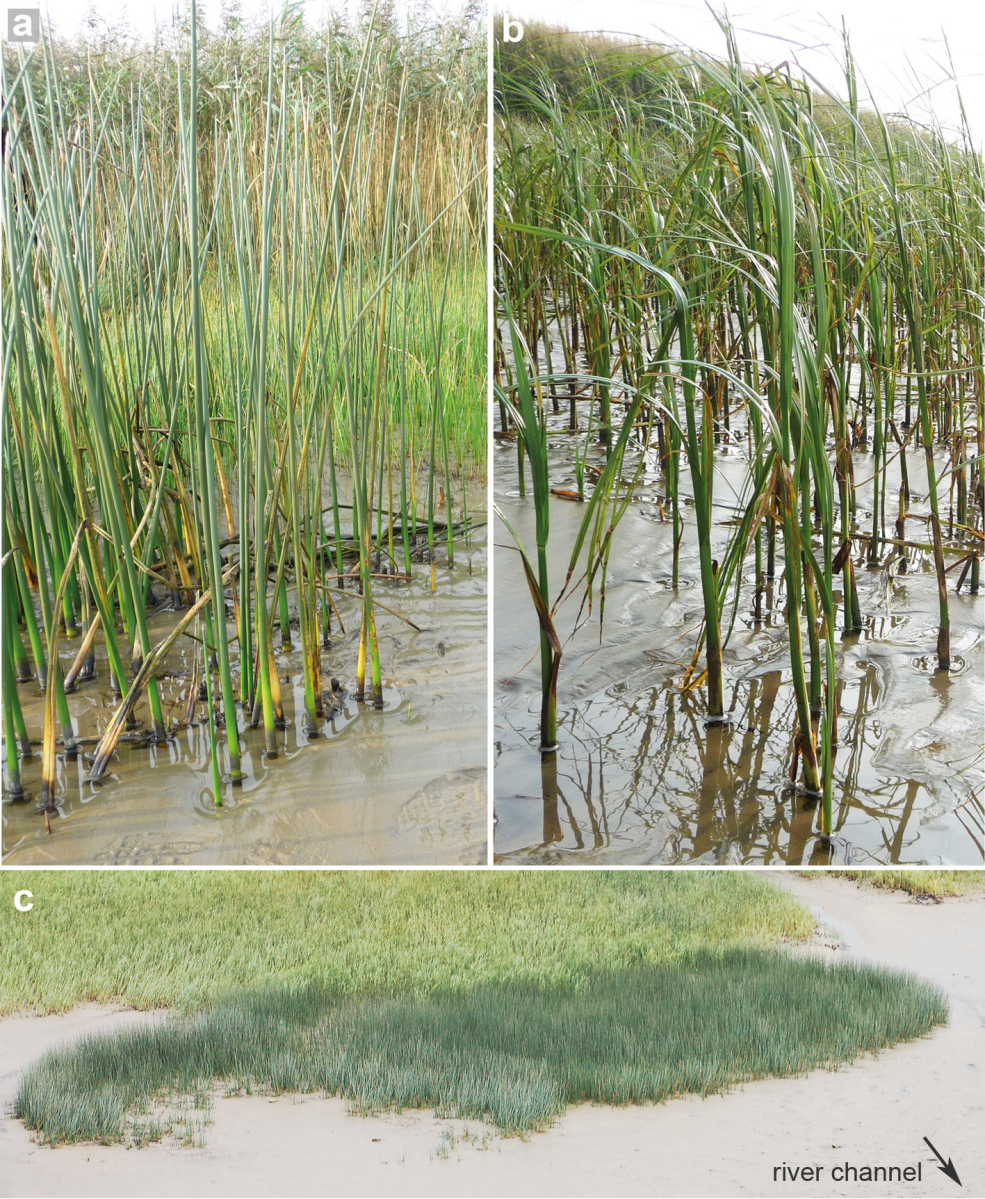
The appearance and zonation of the studied species. a: *S*. *tabernaemontani*, b: *S*. *maritimus* at Hollerwettern (53°50'20"N, 9°21'40"E), c: oblique aerial photo visualizing macrophyte zonation on tidal flats with *S*. *tabernaemontani* in the front (dark green) with the adjacent belt of *S*. *maritimus* (lighter green) at Allwörden (53°49'35"N, 9°19'25"E)

In this paper, we compared wave attenuation as an engineering effect by two plant species that have to withstand similar physical stress conditions on tidal flats. *Scirpus tabernaemontani* (C.C.Gmel.) Palla and *Scirpus maritimus* (L.) Palla are both ubiquitous pioneer species also growing in estuarine tidal marshes along the North Sea (NW Europe). *S*. *tabernaemontani* has an elliptically shaped stem and has no leaves whereas *S*. *maritimus* has a triangular stem and many leaves (Figs 1 and 2). *S*. *tabernaemontani* occurs on lower elevations relative to mean high water (MHW) compared to *S*. *maritimus*. Along the Elbe estuary (Germany), for instance, *S*. *tabernaemontani* is located on average about 50 cm lower than *S*. *maritimus* (mean level in m ± SE relative to MHW, *S*. *tab*.: -1.09 ± 0.03, *n* = 140; *S*. *mar*.: -0.66 ± 0.04, *n* = 140) (S1 Fig). We hypothesize that these realized elevational niches are formed as a result of different strategies (and hence different functional traits) of dealing with hydrodynamic stress. We hypothesize that *S*. *tabernaemontani* has functional traits that allow it to avoid wave induced hydrodynamic stress, whereas *S*. *maritimus* has functional traits allowing to tolerate this hydrodynamic stress. Only regarding the lower relative elevations and disregarding the traits, *S*. *tabernaemontani* would exert a stronger engineering effect exposed to higher water levels and higher waves. In order to compare the traits of the two species and draw conclusions about their effects in estuaries, we performed two laboratory wave flume experiments with individuals of *S*. *tabernaemontani* and *S*. *maritimus* under controlled, standardized water depths and wave heights but with constant elevations.

We investigated how stress resistance affects species functioning with the following questions: (i) does biomass or stem density have a higher ecosystem engineering capacity to attenuate waves, (ii) what are the species responses to wave impact measuring scouring, drag, and bending, (iii) to what extent do species respond to tensile and bending forces, (iv) what are the features of the stem effect traits (strength molecules, shape, cross section) requiring stress resistance, and (v) how much energy is invested in stem material in order to be efficient? We were able to characterize the functional traits in interaction with their EEC regarding the realized niches of *S*. *maritimus* and *S*. *tabernaemontani*.

## Materials and Methods

### Plant material

Approximately 400 pieces of rhizomes were sampled in spring 2012 (April 17^th^ and 23^rd^) for each of the two plant species (Figs 1 and 2). The sampling sites were two brackish marshes. One is located in the Scheldt estuary at Groot Buitenschoor, Belgium (51°21’47"N, 4°14’53"E), where we collected *Scirpus maritimus* (L.). In the Elbe estuary at Hollerwettern, Germany (53°50'20"N, 9°21'40"E), we gathered *Scirpus tabernaemontani* (C. C. Gmel) since this species is not found at Groot Buitenschoor. Access and permission for extraction of plants from the brackish marshes were granted by Natuurpunt (Belgium) and Kreis Steinburg (Germany). We confirm that the field studies did not involve endangered or protected species. For flume exp. 1, 340 rhizome pieces of each species were planted into eight boxes (40 cm, 25 cm, 30 cm) per species and filled with natural sediment from the Scheldt. Continuous marsh vegetation can be simulated by fitting these boxes in two rows into a flume channel at the NIOZ in Yerseke. For detailed properties of the flume see Bouma et al. (2005). Throughout the growing season, *S*. *tabernaemontani* and *S*. *maritimus* developed a stem density of 700 and 600 shoots per m^2^, respectively. For flume exp. 2, we planted 20 rhizomes per species into PVC tubes of 25 cm height and 12 cm diameter lined with plastic bags and filled with the same natural sediment (D_50_ = 0.32 mm) as used in the sediment box of exp. 1. They were grown outside, close to the Scheldt, for three months and irrigated with brackish water (5 g L^-1^ of salt) representing natural field conditions.

### Flume experiment for measuring ecosystem engineering effects (exp. 1) (question i)

Wave attenuation of the two species was measured along a 1.6 m long and 0.6 m wide plant patch in the NIOZ flume. Behind the plant patch, a wave damping rack was installed to avoid wave reflection. The plant patches were prepared for each species with (i) two equal densities and (ii) two equal biomasses. Comparing the same number of stems (density), we were able to demonstrate the effect of biomass. Comparing the same amount of biomass, we analyzed the effect of the unlike morphology. The lower density patch (300 stems m^-2^) was produced by removing randomly selected shoots from the higher density patch (500 stems m^-2^ for *S*. *maritimus*, 800 stems m^-2^ for *S*. *tabernaemontani*), after having first tested it in the flume. The low density is representative of a field situation in spring, whereas the high density represents a field situation in summer. In order to quantify wave attenuation as a function of submerged dry biomass, the submerged biomass per stem was measured on 98 shoots of *S*. *maritimus* and 104 shoots of *S*. *tabernaemontani* that were randomly selected at the end of exp. 1. The lower 32 cm of the stems, which had been inundated during the experiments, were cut off and dried for 72 h at 70°C. The dry biomass per shoot was weighed. Different biomasses were calculated by bootstrapping (random sampling, 10,000 times, checking 99% confidence interval) using the measured total stem density per m^2^. Thereof, the stem density and dry biomass are highly collinear (Pearson correlation 0.94). 300 mg m^-2^ dry biomass corresponds to 200 stems of *S*. *maritimus* and 300 stems of *S*. *tabernaemontani*. 800 mg m^-2^ dry biomass is equal to 500 stems of *S*. *maritimus* and 800 stems of *S*. *tabernaemontani*. Thus, stem density and dry biomass were tested separately.

The waves were measured by calibrated pressure sensors (GE Druck PTX1830) with a sampling frequency of 40 Hz. To be able to deduce wave attenuation from these measurements, we measured waves at two locations: 40 cm in front of the plants and directly behind the plants. In a water depth of 32 cm, the incoming mean wave height (cm ± SE) in front of the plants was 8.4 ± 0.01 (n = 120).

### Flume experiments for measuring plant responses to wave impact (exp. 2) (question ii)

**Fig 3:**
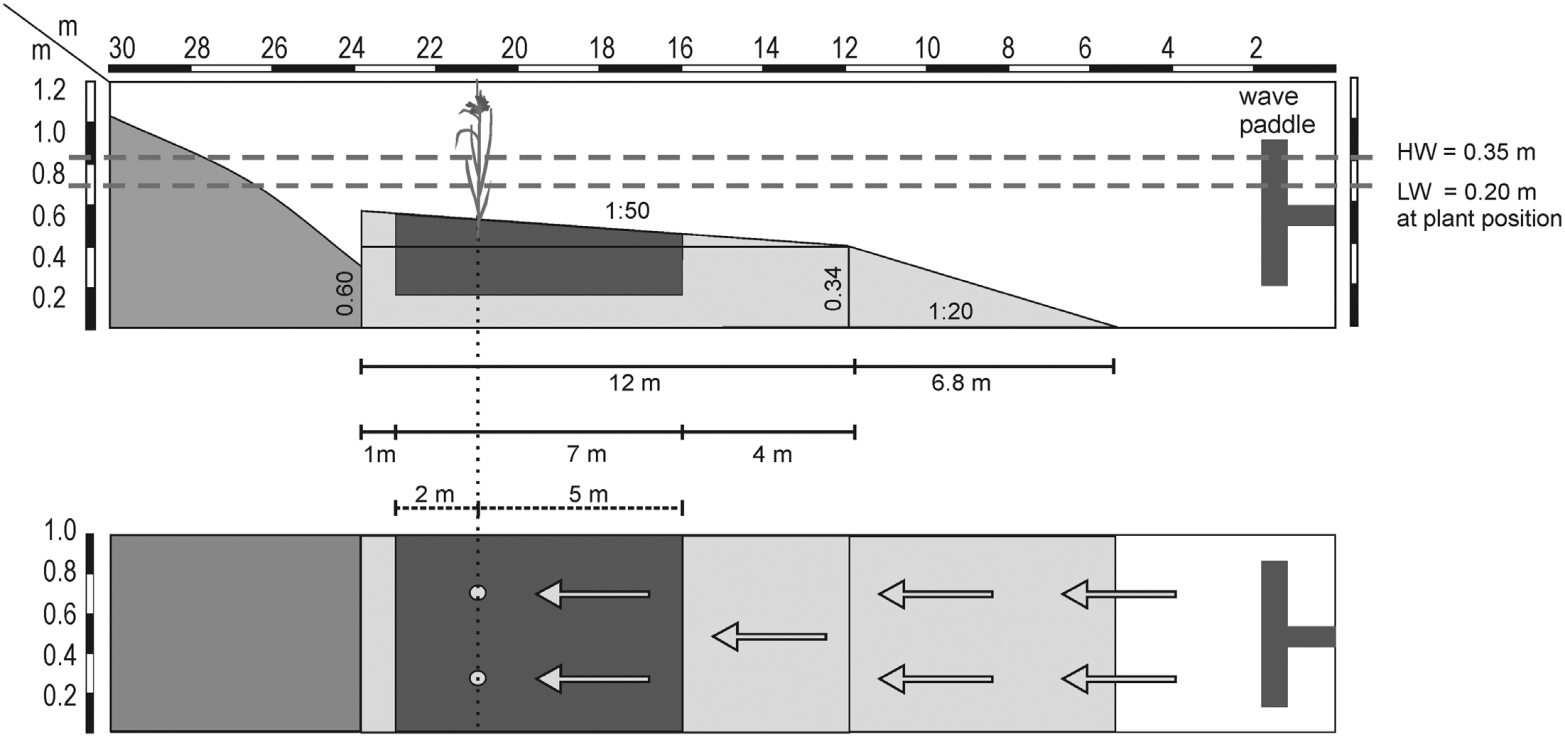
Physical model of wave flume experiments for testing drag force, scouring and bending angle after wave impact of *S*. *tabernaemontani* and *S*. *maritimus*. Top: side view, below: top view. Two plants are situated at the back of the sediment box (dark grey); medium grey: pebble stone beach for wave absorption, light grey: concrete bottom slope; HW = high water depth, LW = low water depth.

The full-scale experiments were performed in an engineering wave flume facility at Ghent University. The experimental wave flume (Fig 3) consisted of subsequent slope sections with a rough sand-like concrete surface. First, the transition slope facing the wave paddle was necessary in order not to lose too much wave energy over a longer gentle slope. The second slope with the test section represents a gently inclined estuarine tidal flat colonized with pioneer vegetation dominated by *S*. *tabernaemontani* and *S*. *maritimus*. The pebble stone beach at the rear of the flume absorbed the waves and avoided wave reflection from the back.

The integrated sand box 0.3 m in depth was filled with natural sand from the Scheldt estuary (D_50_ = 0.32 mm). Acting as the first plant row of a marsh edge, two plants were transplanted at the back of the sand box for each test. The tubes were removed and the plastic bag folded deeply downwards into the surrounding sediment box. With this method, plants could be transplanted with their anchored root system and no sediment border for undesirable side effects between the transplanted plant and the sediment of the flume sediment box. For producing and measuring a representative scouring of one stem, all clonally grown stems, except for one, were removed from the transplanted plant by cutting them off as deep as possible below the sediment surface. Plant properties such as the height of stem and the stem diameter were measured before the runs of exp. 2 to record the initial plant-morphological status. The properties of these two species were not significantly different. The mean stem height (cm ± SE) of *S*. *maritimus* is 102 ± 4.7 and of *S*. *tabernaemontani* 114 ± 3.4. The mean stem diameters (mm ± SE) are 8.2 ± 0.4 (*S*. *maritimus*) and 8.9 ± 0.4 (*S*. *tabernaemontani*).

We tested two water depths (of 20 cm and 35 cm at plant position) with a constant wave period of 2 s for ten individuals of each of the two plant species. The two water depths simulate wave impact at different moments in the tidal cycle, because the mean tidal range (2001–2010) in the Elbe estuary, for instance, varies between 2.8 m (gauge Brokdorf, 53°51'53"N 9°19'13"E) and 3.6 m (gauge St. Pauli, 53°32'45"N 9°57'33"E). The 2 s wave period was chosen to simulate common natural wind waves in the Elbe estuary [39]. Each test run consisted of 200 waves and for each of them two replicate individuals were transplanted next to each other with an exact distance of 0.33 m between the two stems as well as between a stem and the flume margin. The surface of the sand slope was flattened to an initial slope of 1:50 before each test. Using resistance wave gauges (sampling frequency 40 Hz), waves were measured at the paddle and on the test section for each of the test runs. Wave height (cm ± SE) at the paddle was set to 17 ± 0 (*n* = 20). The waves were transformed on the slope and reached a mean wave height of 9 ± 0 (*n* = 10) for 20 cm water depth (broken waves) and 18 ± 0 (*n* = 10) for 35 cm water depth (unbroken waves) at the location of the plants.

We measured plant characteristics (height, number of leaves, leaf length, and stem diameter 3 cm above the sediment surface), drag forces acting on the plants during wave impact, scouring depths and volumes around the stems [40], as well as bending angle before and after each test run. Drag forces were measured by a bimetal calibrated for measurements in N (Versluys’s drag instrument developed by Ghent University, Dept. of Civil Engineering, Belgium). Peak drag forces were extracted and averaged per plant and test run. Number of leaves, leaf length and stem diameter as well as drag force and effective water level of each condition (i.e. still water level + significant wave amplitude) were used to calculate the drag per wet frontal plant area. After cutting the plant, the scoured sediment surface around the stem, plus a reference section between the plants without interference of a stem, were scanned by a laser scanner (EProfiler developed by Aalborg University, Hydraulic & Coastal Engineering Group, Denmark) (± 1 mm). In order to quantify scouring depth and volume as measures of scour vulnerary, the scouring data were analyzed with the Hydrology Toolbox of Esri ArcGIS.

### Measurements of biomechanical plant traits (question iii)

The biomechanical properties of the two plant species studied were measured on the basal parts of the stems of 20 individuals per species that were not subjected to any experiment. Bending and tensile tests were performed with a universal testing machine (Instron 5942, Canton, MA, USA). The bending test was conducted on the most basal part of the stems and the pulling test on a slightly higher section. For each test, the stem fragments were 10 cm long. For each sample, we measured the dimensions of the cross section using a digital caliper (± 0.02 mm) at three different points along the sample: height and width for triangular cross-sections (*S*. *maritimus*) and axes for the elliptical cross-sections (*S*. *tabernaemontani*). For the bending tests, we performed three-point bending tests, consisting of a force applied at a constant rate of 10 mm min^-1^ to the midpoint of a sample placed on a support. For the tensile tests, the stem fragments were clamped into the jaws of the testing machine and a constant extension rate of 5 mm min^-1^ was applied to the upper jaw until they broke.

The bending tests were used to calculate Young's modulus, the second moment of area, and the flexural stiffness. Young’s modulus (*E* in Pa) quantifies the material stiffness and is calculated as the slope of the stress-strain curve in the elastic deformation region. The second moment of area (*I* in m^4^) accounts for the effect of the cross sectional geometry of a structure on its bending stress. It was calculated depending on the geometry of the cross section by using the equation: $I=bh^{3}\frac{1}{36}$, where *b* and *h* are the base and height of the cross section for *S*. *maritimus* and $I=\frac{\pi }{4}ac^{3}$, where *a* and *c* are the shorter and longer axes of the cross section for *S*. *tabernaemontani*. The flexural stiffness (*EI* in N m^2^) quantifies the stiffness of the stem fragment and was calculated by multiplying *E* and *I*. In many cases there was no real breakage (but rather a buckling of the stem fragment), because the stems were too flexible. The tensile tests were used to calculate the breaking force and the tensile strength. The breaking force (N) is the force at which the plant fragment breaks when exposed to tensile forces (e.g. hydrodynamic forces). The tensile strength (N m^-2^) is the breaking force corrected by the cross sectional area of the fragment.

### Analysis of plant strength (question iv)

Ten individual *S*. *tabernaemontani* and *S*. *maritimus* stems that were not subjected to any experiment, were split into the basal 30 cm and the remaining upper stem part of various lengths. These parts were dried at 70°C, ground, and weighed. Biogenic silica (BSi) was extracted from 25 mg dry plant material of each individual by incubation in a 0.1 M Na_2_CO_3_ mixture at 80°C for 4 h (DeMaster, 1981). The extracted and dissolved silica was analyzed on an ICP-OES spectrometer (Skalar, The Netherlands). To determine the cellulose and lignin contents, the Van Soest method [41] was used. We analyzed the contents of the strength molecules silica, lignin and cellulose in two stem parts of the two species performing a two-way ANOVA with post hoc Tukey’s HSD test for each molecule.

### Calculation of cost efficiency (question v)

The cost involving stem strengthening was determined by the biomass produced, the amount of strengthening molecules (cellulose and lignin) and stem shape. We recorded the dry biomass after exp. 1. was conducted.

We used the cost energy values of lignin and cellulose from Jung et al. [42]: 29,128 kJ kg^-1^ for lignin and 16,747 kJ kg^-1^ for cellulose. These were multiplied with the mean concentration of these respective molecules as found by our analysis of the basal stems. The gross energy is the sum of energy invested in cellulose and lignin. The cross-sectional area of *S*. *tabernaemontani* (elliptical stem) and *S*. *maritimus* (triangular stem) was measured with a digital calliper (see objective iii). Finally, the mean dry biomass was multiplied with the gross energy for each species and normalized by the stem cross-sectional area.

## Results

### Ecosystem engineering effects (question i)

**Fig 4:**
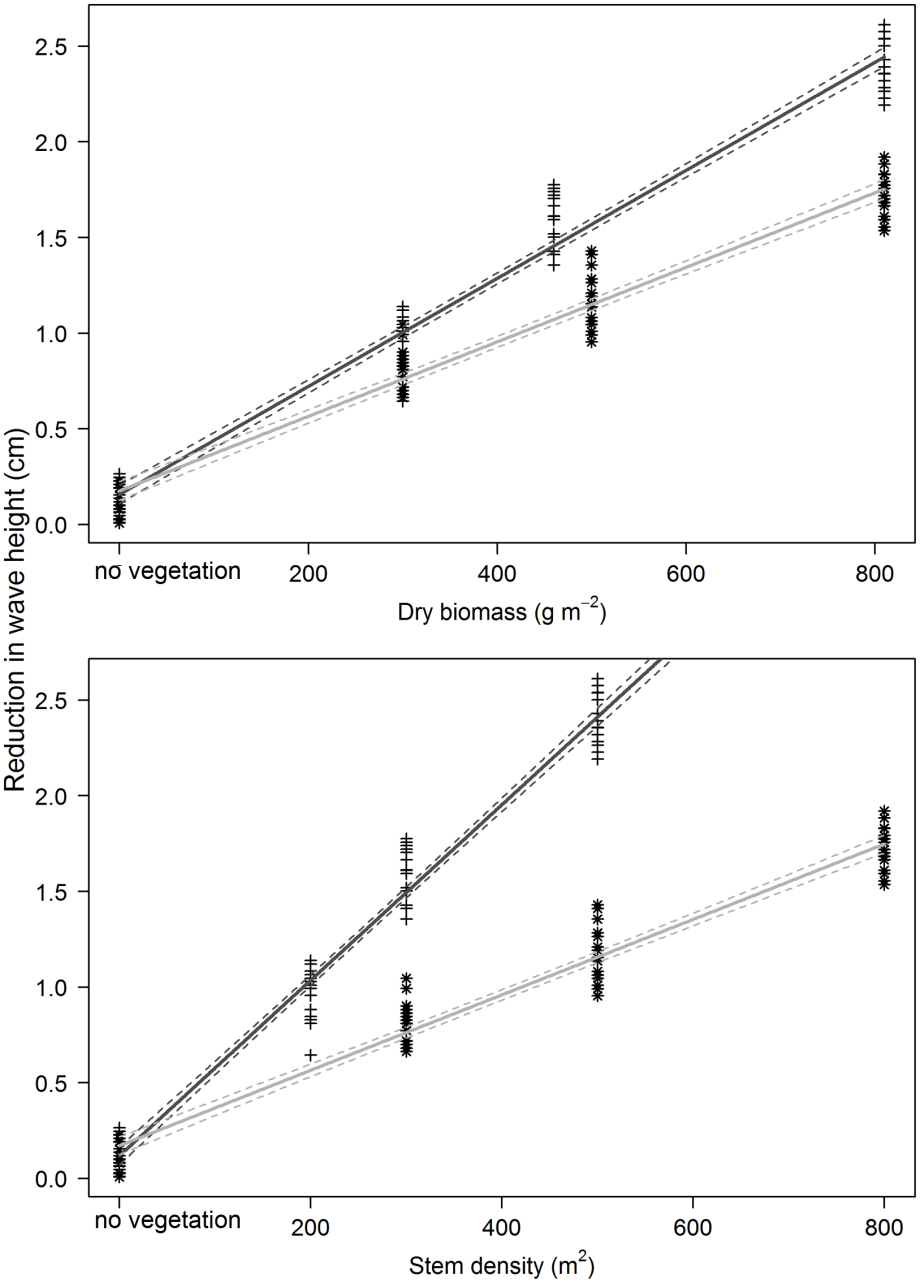
Wave attenuation behind a patch 1.6 m length, in relation to stem densities and in relation to dry submerged biomass for the species *S*. *maritimus* (+) and *S*. *tabernaemontani* (*). The wave attenuation was well explained by the species’ biomass (F_3,156_ = 1645, ρ < 0.001, R^2^_adj_ = 0.97) as well as by the species’ stem densities (F_3,156_ = 1857, ρ < 0.001, R^2^_adj_ = 0.97) using ANCOVA. The significantly different regression slopes exhibited the unlike species’ effect of wave attenuation (biomass: Reduction_wave_ = 0.157 + 0.003 biomass for *S*. *maritimus*, Reduction_wave_ = 0.176 + 0.002 biomass for *S*. *tabernaemontani*; stem density: Reduction_wave_ = 0.119 + 0.005 stem density for *S*. *maritimus*, Reduction_wave_ = 0.171 + 0.002 stem density for *S*. *tabernaemontani)*. Lines represent the linear regression with 95%-confidence intervals (dotted lines), the regression assumptions were checked with diagnostic plots with positive results, no vegetation = the control wave runs.

Both species significantly attenuated waves (Fig 4). However, the patch of *S*. *maritimus* showed a stronger reduction in wave height. The differences in wave attenuation between the species were larger with equal stem density than with equal dry biomass. Although the relation strength was very high (R^2^ = 0.97) for both predictors, stem density and dry biomass, the wave attenuation by the stem density of *S*. *maritimus* featured a higher effect due to its higher regression slope. The wave attenuation by the stem density of *S*. *tabernaemontani* was coincident with wave attenuation by its dry biomass (Fig 4).

### Plant responses to wave impact (question ii)

The mean drag force on *S*. *maritimus* was twice as high (20 cm: 1.3 N, 35 cm: 2.7 N) as the mean drag force on *S*. *tabernaemontani* (20 cm: 0.6 N, 35 cm: 1.2 N). This difference disappeared when normalizing the parameter drag force to drag force per wet frontal plant area. Species and water depth interacted significantly: At a water depth of 20 cm, *S*. *maritimus* (289 N m^-2^) experienced nearly 100 N m^-2^ more drag force than *S*. *tabernaemontani* (197 N m^-2^). At a water depth of 35 cm, the differences in drag force per wet frontal plant area were less than 15 N m^-2^ (*S*. *tabernaemontani*: 261 N m^-2^, *S*. *maritimus*: 275 N m^-2^) and were no longer significant.

**Fig 5:**
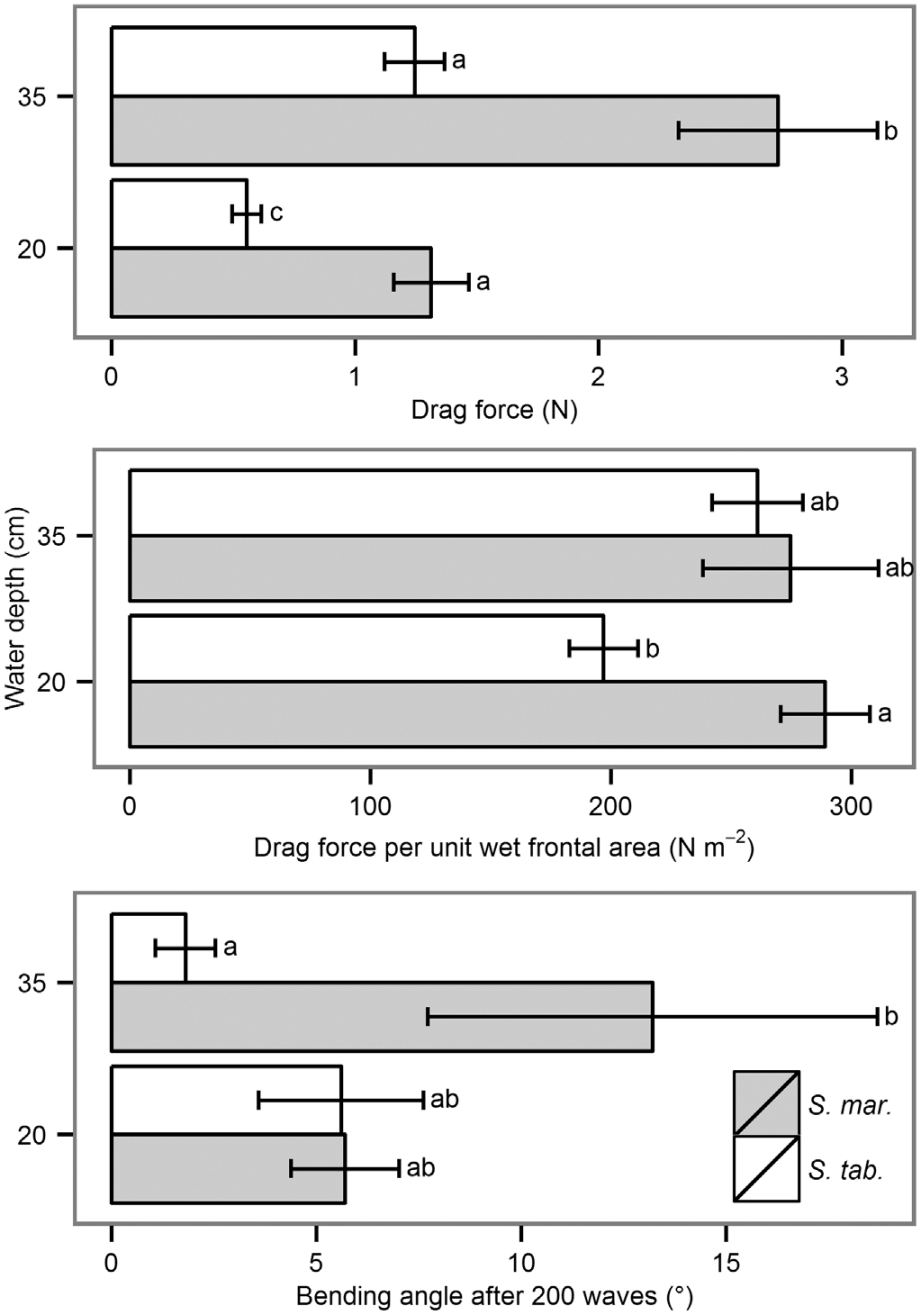
Mean values of plant responses to wave impact with standard error (*n* = 10) for *S*. *maritimus* (*S*. *mar*.) vs. *S*. *tabernaemontani* (*S*. *tab*.). For ANOVA, the response variables were transformed by the natural logarithms. The absolute drag force differed significantly between species (F1,36 = 41.1, ρ < 0.001) and water depths (F1,36 = 36.5, ρ < 0.001). The drag force per unit of wet frontal area showed a significant interaction between species and water depth (F3,36 = 4.8, ρ = 0.035). Regarding the bending angle, the water depth of 35 cm demonstrated a significant difference between the species (F3,36 = 4.4, ρ = 0.044). Different letters show significant differences, significance level is α < 0.05.

After 200 waves, both species bent very little at low water depth, whereas *S*. *maritimus* exhibited significant bending at high water depth (Fig 5). The species did not differ in scouring volume or depth (data not shown). Furthermore, the highest observed absolute values (1.1 cm scouring depth and 19 cm^3^ scouring volume) were insufficient for uprooting.

### Plant resistance (questions iii and iv)

**Fig 6:**
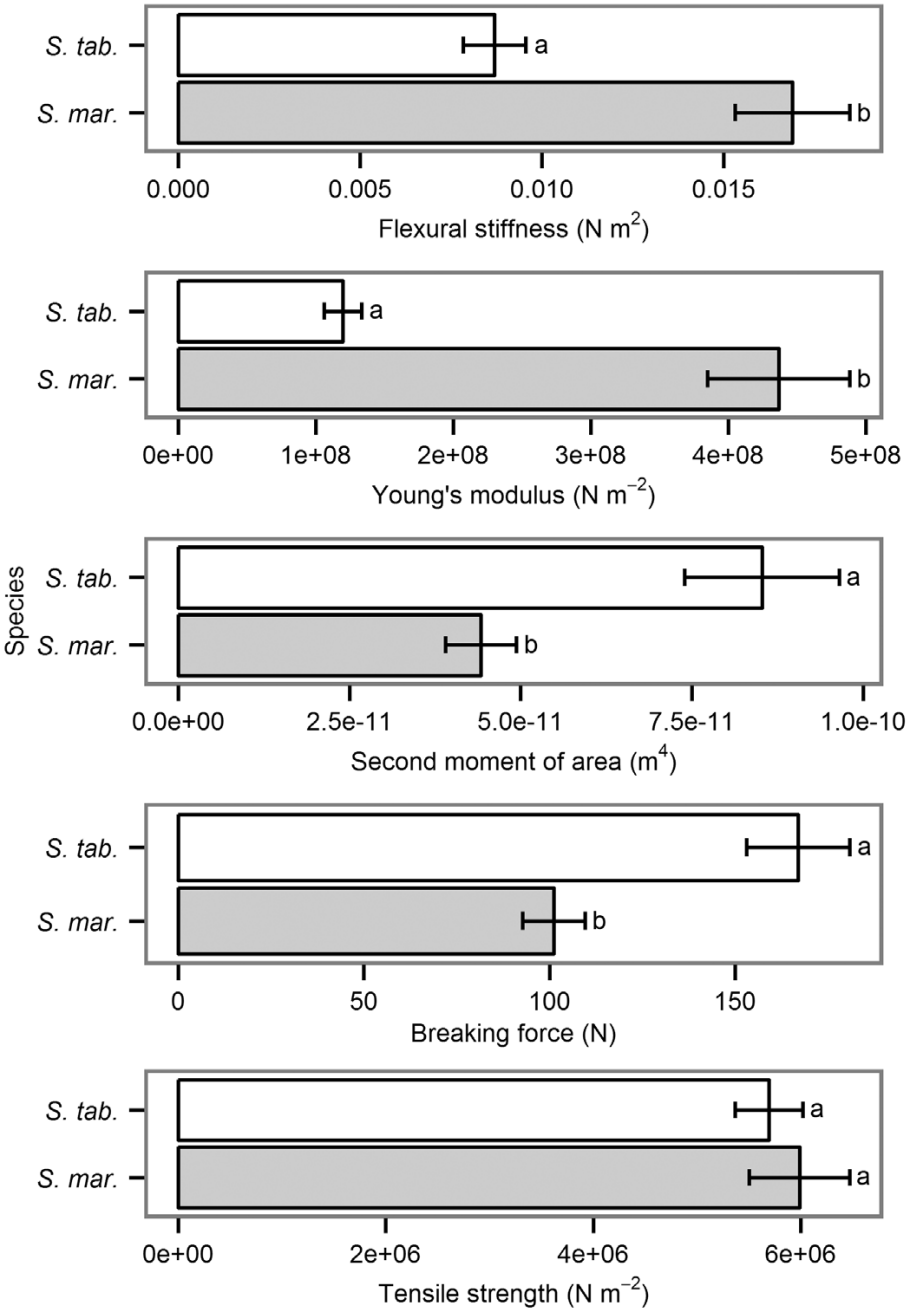
Mean values of plant responses to tensile and bending forces with standard error for *S*. *maritimus* (*S*. *mar*., *n* = 19) vs. for S. *tabernaemontani* (*S*. *tab*., *n* = 20). For ANOVA, the response variables were transformed by the natural logarithms except for tensile strength. These data showed variance homoscedasticity of the residuals without transformation. The two species differed significantly in Young’s modulus (F1, 37 = 56.3, ρ < 0.001), in second moment of area (F1, 37 = 14.1, ρ < 0.001), in flexural stiffness (F1, 37 = 13.9, ρ < 0.001), and in breaking force (F1, 38 = 16.9, ρ < 0.001), but not in tensile strength. Different letters show significant differences, significance level is α < 0.05.

Both species differed in regards to biomechanical properties but for tensile strength: e.g. Young’s modulus was four times higher for *S*. *maritimus* (4.4 10^8^ N m^-2^) than for *S*. *tabernaemontani* (1.2 10^8^ N m^-2^) indicating stiffer tissues for *S*. *maritimus*, whereas the second moment of area was nearly two times higher for *S*. *tabernaemontani* (8.5 10^−11^ m^4^), than for *S*. *maritimus* (4.4 10^−11^ m^4^). The product of these two parameters resulted in the flexural stiffness being significantly different between the two species (Fig 6): with 0.017 N m^2^, stems of *S*. *maritimus* were about twice as stiff as the ones of *S*. *tabernaemontani* (0.009 N m^2^). Regarding the tensile properties, the breaking force of the stems significantly differed between the two species (167 N for of *S*. *tabernaemontani* vs. 101 N for *S*. *maritimus*, Fig 6). However, they did not differ significantly in tensile strength, i.e. tensile force corrected for cross-sectional area (Fig 6), which indicated that the tissues (material) of *S*. *tabernaemontani* were not more resistant to tensile force than those of *S*. *maritimus*.

**Fig 7:**
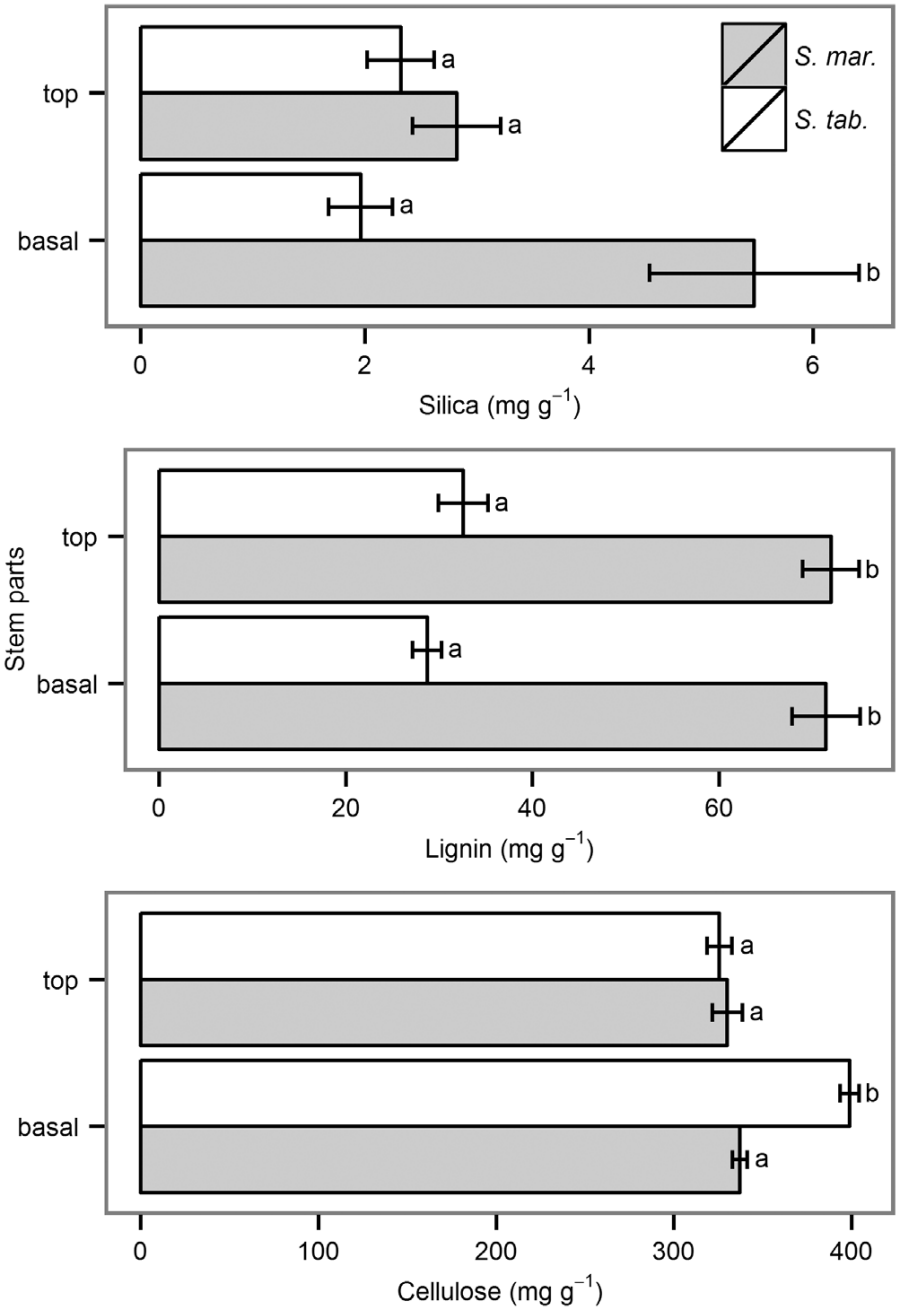
Mean effect traits with standard errors (*n* = 10) for the species *S*. *maritimus* (*S*. *mar*.) and *S*. *tabernaemontani* (*S*. *tab*.). For ANOVA, silica data were transformed by the natural logarithms to insure the variance homoscedasticity of the residuals. Lignin and cellulose data showed variance homoscedasticity of the residuals also without transformation. The stem parts interacted significantly with the species in the silica content (F_3,36_ = 5.3, ρ = 0.028) as well as in the cellulose content (F_3,36_ = 26.4, ρ < 0.001). The species differed significantly in the lignin content (F_3,36_ = 211.5, ρ < 0.001). Different letters show significant difference, significance level is α < 0.05.

Regarding the cell wall, *S*. *maritimus* had an overall higher content of silica and lignin compared to *S*. *tabernaemontani*. Unlike the basal stem parts, the upper stem parts showed no significant difference in silica content between the two species (Fig 7). Furthermore, the basal stem parts of *S*. *maritimus* had a significantly higher silica content than the upper stem parts (5.5 mg g^-1^ and 2.8 mg g^-1^ respectively), while *S*. *tabernaemontani* had no significant difference between basal and upper stem parts. The lignin content differed significantly between the species: *S*. *maritimus* had more than twice the lignin contents of *S*. *tabernaemontani* (total mean of 71.7 mg g^-1^ and 30.6 mg g^-1^ respectively). Stem parts within both species did not show any differences. While the cellulose content in the upper stem parts of both species was almost identical, the basal stem parts of *S*. *tabernaemontani* contained clearly more cellulose (397.0 mg g^-1^) than the basal parts of *S*. *maritimus* (337.0 mg g^-1^) and the upper stem parts.

### Cost efficiency (question v)

For building strength molecules, the gross energy investment into a 32 cm basal stem parts of *S*. *tabernaemontani* was 7515 kJ kg^-1^ dry matter, whereas the gross energy investment into that of *S*. *maritimus* amounted to 7725 kJ kg^-1^ dry matter. This mean that *S*. *tabernaemontani* had to invest 8 kJ into its basal stem part, while *S*. *maritimus* needed 11 kJ, making S. *tabernaemontani* 31% more efficient than *S*. *maritimus*. This difference could be attributed mainly to the differences in biomass and strength molecules. Dry biomass weight (g ± SE) of the basal stem part of *S*. *tabernaemontani* was 1.0 ± 0.04 (*n* = 104), while this part of *S*. *maritimus* weighed 1.4 ± 0.06 (*n* = 98). Cellulose and lignin concentrations were also significantly different for both species (Fig 7). These data were corrected for the stem cross-sectional area. The stem cross-sectional area was a product of diameter and stem shape. The stem diameter between the two species did not differ significantly, but *S*. *tabernaemontani* had a significantly larger cross-sectional stem area. The mean stem cross-sectional area (mm^2^ ± SE) of *S*. *tabernaemontani*, which had an oval shape, was 33.2 ± 2.3 (*n* = 19), whereas *S*. *maritimus*, which had a triangular shape, had a mean stem area of only 26.3 ± 1.3 (*n* = 20). Corrected for the stem cross-sectional area, the gross energy invested by *S*. *maritimus* was with 0.42 kJ mm^-2^ twice as high as of *S*. *tabernaemontani* (0.21 kJ mm^-2^).

## Discussion

**Fig 8:**
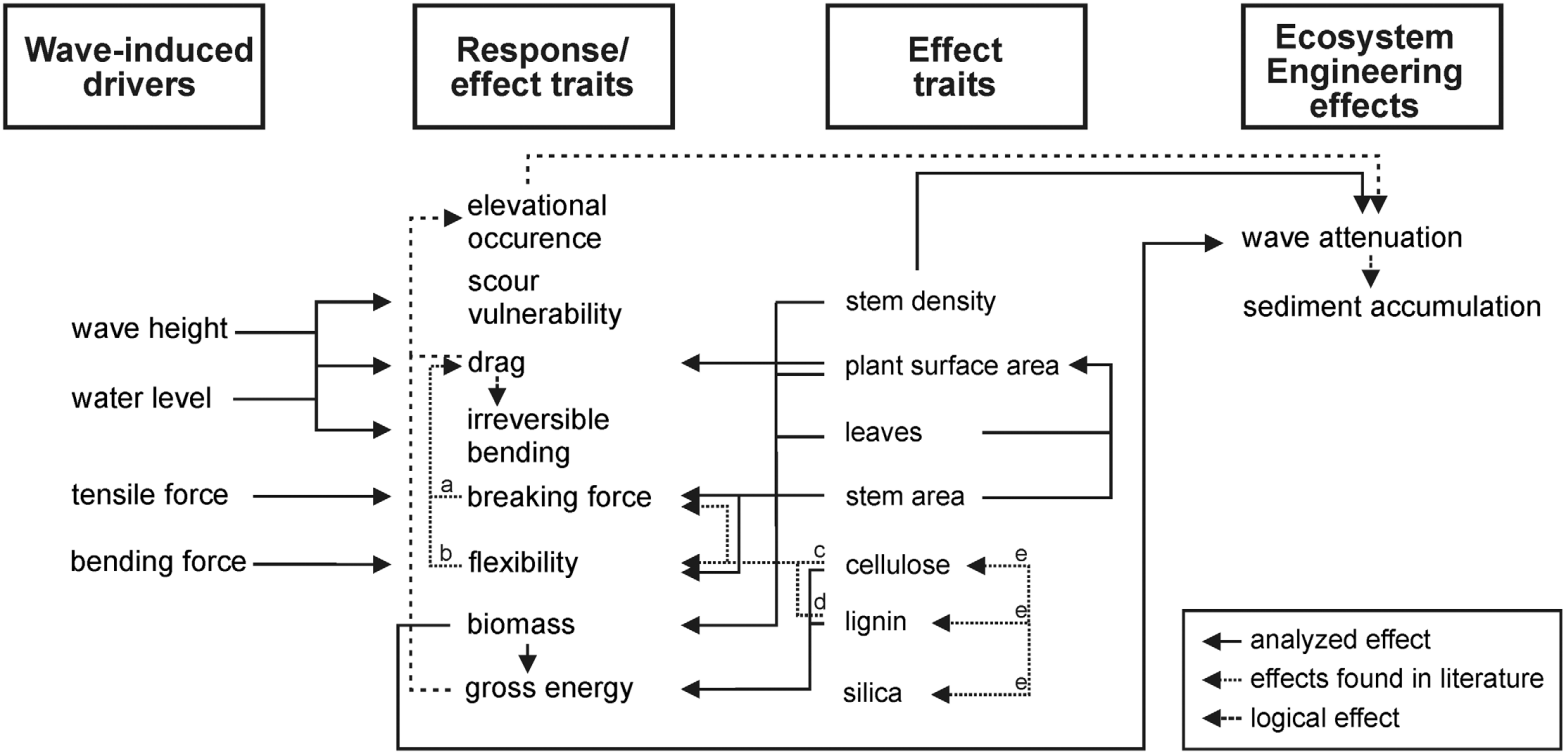
Conceptual framework of the relationships between traits which respond to the environment drivers (response traits) and traits which determine the effects of plants and thus the effect of ecosystem engineering (effect traits) in a wave-exposed intertidal habitat. Thus, the double-heading ‘response/ effect traits’ classify traits with both properties: They respond to the wave-induced drivers and at the same time they determine the effect of wave attenuation. Solid arrows represent the effects which we analyzed, whereas dotted arrows stand for effects we found in literature (a:[33], b: [18–20], c: [37] d: [38], e: [36]), and dashed arrows show the effects which we hypothesize from our results.

The link between the ecosystem engineering capacity (EEC) of plants and their functional traits has been poorly studied so far [20, 43, 44]. To our knowledge this study is the first one to verify the EEC by demonstrating the relations of response and effect traits regarding the effect of wave attenuation (Fig 8). *S*. *tabernaemontani* and *S*. *maritimus* can be seen as examples of plant ecosystem engineers growing on estuarine low marshes, where wave attenuation within the vegetation leads to sedimentation and a change in the environment. In full-scale wave flume experiments, the species exhibit clear dissimilarities in their response traits, which are also reflected in the analyses of their effect traits. Generally, a higher content of strength molecules was built into the basal stem part of both species (except for lignin). The strength of the base functions as ‘mechanical fuse’ protecting the root system [45], and ‘carrying’ the rest of the upper stem part. Yet it is likely that exactly this stem strength has important effects on (i) the experienced stress, and thus on (ii) the capacity for wave attenuation.

At the water depth of 20 cm, drag forces on *S*. *tabernaemontani* and *S*. *maritimus* are significantly different (Fig 5). The reason is their discrepancy in outer shape (e.g. leaf number), and the higher tissue rigidity [46]. The clear difference, however disappears at the 35 cm depth due to the fact that more leaves of *S*. *maritimus* are submerged increasing the frontal area drastically [47]. The significant higher bending angle of *S*. *maritimus* in contrast to *S*. *tabernaemontani* at the water depth of 35 cm is an inextricable consequence (Fig 8). Bending can be considered as the mechanism leading to toppling [40] and toppling is the determinant process that causes mortality of the plants, and hence failure of plant survival on the intertidal flats, but also for mangroves [48]. Furthermore, the irreversible bending of plants in our experiment only results from drag, but not from scouring. Consequently, the species’ survival performance, and with it the safety of species’ functioning is implied to be only determined by the amount of drag force that the plants experience.

The higher drag force which *S*. *maritimus* suffers in contrast to *S*. *tabernaemontani* might be an explanation why it does not grow at lower elevations. Nevertheless, it also benefits from the advantage of wave attenuation. The higher effect explained by stem density compared to the effect explained by biomass regarding *S*. *maritimus* states biomass is more relevant than the species morphology which points to the plant surface area, an effect trait of biomass on which friction can be created. Thus, the plant surface area appears to be the prime driver for significant wave attenuation [49]. The presence of leaves, the roughness and stiffness of stems are relevant factors for effective wave attenuation (besides stem deflection [15]), and also points towards being responsible for the significant differences between *S*. *maritimus* (with leaves) and *S*. *tabernaemontani* (leafless). Thus, besides the plant surface area and stem density, the species’ functioning in terms of ecosystem engineering indicate being directly affected by the degree of tissue rigidity.

Our findings fit the classical conceptual model of the avoidance-tolerance trade-off [32, 33]. In contrast to *S*. *maritimus*, *S*. *tabernaemontani* has more traits yielding stress avoidance such as low lignin and silica content (Table 1), which results in higher flexibility (i.e. lower stiffness) [36] and hence a reduction of experienced drag forces [36, 40, 50]. The lighter biomass also refers to the reduction of drag forces. However, *S*. *tabernaemontani* has also a higher cross-sectional stem area that can resist higher tensile forces. Contrary to *S*. *tabernaemontani*, *S*. *maritimus* has more tolerating traits, such as high lignin and silica content, which likely results in higher experienced drag forces and higher material costs. Contrary to the stiffness, the differences in tensile strength of the two species are not substantiated by the composition of the cell walls. The stem cross-sectional area is the main driving factor here for the significant differences in breaking force. The stem cross section of *S*. *tabernaemontani* is larger, leading to higher resistance to breaking compared to the stem of *S*. *maritimus*. Despite a larger cross sectional area, *S*. *tabernaemontani* has a lower material investment than *S*. *maritimus* per unit stem length, because of its lower biomass weight and lesser kilojoule costs for cellulose than for lignin. Thus, this pattern indicates a trade-off between concentrations of strength molecules in the cell walls, stem shape, biomass, and energy investment: The lower the material investment, the larger the cross sectional area being more resistant to breaking force.

**Table 1:** The elevational habitat responses linked with the analyzed traits and the contrasting findings between *S*. *maritimus* and *S*. *tabernaemontani* which result in two strategies of stress resistance.

Traits	*S*. *maritimus*	*S*. *tabernaemontani*
elevational occurrence	+	-
scour vulnerability	±	±
drag	+	-
irreversible bending	+	-
breaking force	-	+
flexibility	-	+
biomass	+	-
gross energy	+	-
stem density	±	±
plant surface area	+	-
leaves	+	-
stem area	-	+
cellulose	-	+
lignin	+	-
silica	+	-
wave attenuation	+	-
**Sum**	**Tolerance**	**Avoidance**
	**Strategy of stress resistance**	

+ stands for traits which are more or higher as compared to the traits of the other species.

- defines the trait specification which is less or smaller as compared to the traits of the other species.

± means no trait difference.

All trait specifications of *S*. *maritimus* reflect the tolerance strategy, whereas all trait specifications of *S*. *tabernaemontani* mirror the avoidance strategy.

### Why do plant species need a different level of ecosystem engineering capacity, when they live in the same habitat?

The results of the functional traits likely explain the clear elevational occurrence of *S*. *tabernaemontani* and *S*. *maritimus* (Fig 2c). Our results support the hypothesis that *S*. *tabernaemontani* has more avoiding traits than *S*. *maritimus* (Table 1), which enables it to withstand the higher hydrodynamic load that is typically found at lower marsh elevations where water levels are higher. Thus, the low cost efficiency and limited material investments of *S*. *tabernaemontani* may result from a high risk of failure due to the exposure to strong hydrodynamic forces. Diminishing the experienced drag force due to its stem flexibility, is likely to reduce its failure. Furthermore the lower marsh elevations with longer inundation durations implicate that *S*. *tabernaemontani* performs the function of wave attenuation more regularly than *S*. *maritimus*. Although the traits of *S*. *tabernaemontani* have a less powerful effect on wave attenuation than *S*. *maritimus*, it is still a significant effect, especially at high stem densities. By forming a sort of barrier that attenuates the waves to a certain extent, *S*. *tabernaemontani* may provide *S*. *maritimus* a suitable habitat further up the marsh (interspecific facilitation). This ecological engineering is likely to be essential in cases when hydrodynamic conditions are too harsh for the establishment of *S*. *maritimus* by itself due to its high experienced drag force.

*S*. *maritimus*, on the contrary, is more capable of attenuating waves, which is assumed to be a benefit for its survival on the upper side of the marsh edge where much lower water levels are present. It probably creates a more benign habitat through sediment accretion [51, 52] and its stronger EEC on higher elevation may empower *S*. *maritimus* to easily outcompete *S*. *tabernaemontani* indicating marsh zonation as a product of dominant plants monopolizing physically gentle habitats and pushing off subordinate plants to physically harsh habitats [53]. To ensure this hypothesis, experiments on the species’ competition abilities in interaction with hydrodynamic forces are required. Our study could demonstrate that *S*. *tabernaemontani* and *S*. *maritimus* are both important ecosystem engineers with unlike EEC due to contrasting functional traits and distinct elevational distribution.

## Conclusion

As a clear example of ecosystem engineering provided by *S*. *tabernaemontani* and *S*. *maritimus*, the effect of biomass verified by stem density as a strong predictor for wave attenuation seem to be more relevant for the engineering effect of emergent macrophytes having leaves than species morphology. *S*. *tabernaemontani* predominantly exhibits stress avoiding traits which facilitates its survival in physical stressful environments. In contrast, *S*. *maritimus* has mainly stress tolerating traits, whose response-effect relations lead to a higher effect of wave attenuation. This signifies that morphological traits of emergent macrophytes are strongly intertwined with their experienced stress (responses) by hydrodynamic forces, their stress resisting capacity (effects), and their ecosystem engineering effects which confirm therefore to be appropriate measures for the capacity of ecosystem engineering [18, 20]. Our study could be a representative example for illustrating the trait relations between environmental responses and ecosystem effects and how these traits overlap [22, 24]. Functional traits seem to be directly balanced to habitat characteristics such as the exposure to wave load and where realized elevational niches are likely formed as a result of different strategies to deal with this hydrodynamic stress. To what extent trait-habitat balance will be maintained remains a focal point of research, especially in a world where tidal ecosystems are subjected to processes of anthropogenic modification and apparent sea level rise [54].

## Supporting Information

S1 Fig Mean elevation relative to mean high water (MHW) including standard error, where *Scirpus tabernaemontani* (*S*. *tab*., n = 140) and *Scirpus maritimus* (*S*. *mar*., n = 140) are situated at the Elbe estuary. The point dataset was randomly sampled from a digital vegetation map (scale: 1: 5000) combined with officially certified digital elevation data, both made in the year 2010. Significance (α) was tested by the Kruskal-Wallis rank sum test. Different letters show significant difference, significance level is α < 0.01.
(TIF)

S1 File Silinski A, Heuner M, Schoelynck J, Puijalon S, Schröder U, et al. (2015) Effects of wind waves versus ship waves on tidal marsh plants: a flume study on different life stages of Scirpus maritimus. PLoS ONE 10: e0118687.
(PDF)
