# Correction: Ecosystem Engineering by Plants on Wave-Exposed Intertidal Flats Is Governed by Relationships between Effect and Response Traits

**DOI:** 10.1371/journal.pone.0171364

**Published:** 2017-01-26

**Authors:** Maike Heuner, Alexandra Silinski, Jonas Schoelynck, Tjeerd J. Bouma, Sara Puijalon, Peter Troch, Elmar Fuchs, Boris Schröder, Uwe Schröder, Patrick Meire, Stijn Temmerman

S1 Fig appears incorrectly in the published article. Please view the correct S1 Fig below.

## Supporting Information

S1 Fig Mean elevation relative to mean high water (MHW) including standard error, where Scirpus tabernaemontani (S. tab., n = 140) and Scirpus maritimus (S. mar., n = 140) are situated at the Elbe estuary. The point dataset was randomly sampled from a digital vegetation map (scale: 1: 5000) combined with officially certified digital elevation data, both made in the year 2010. Significance (α) was tested by the Kruskal-Wallis rank sum test. Different letters show significant difference, significance level is α < 0.01.
(TIF)
