# Supplementary figures and images for: Ecosystem Engineering by Plants on Wave-Exposed Intertidal Flats Is Governed by Relationships between Effect and Response Traits
Source: PLoS One. 2015 Sep 14;10(9):e0138086. doi: 10.1371/journal.pone.0138086 (PMC4569080; doi:10.1371/journal.pone.0138086)

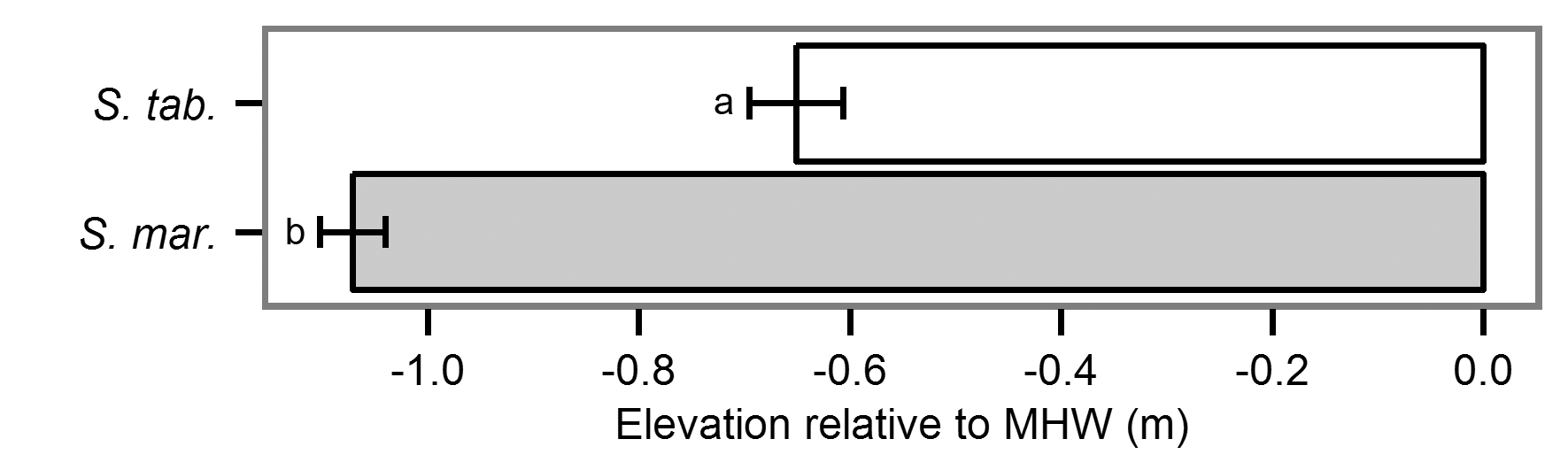

Supplement: S1 Fig — The point dataset was randomly sampled from a digital vegetation map (scale: 1: 5000) combined with officially certified digital elevation data, both made in the year 2010. Significance (α) was tested by the Kruskal-Wallis rank sum test. Different letters show significant difference, significance level is α < 0.01. (TIF) [file pone.0138086.s001.tif]
